# Health Equity and Worker Justice in Temporary Staffing: The Illinois Case

**DOI:** 10.3390/ijerph19095112

**Published:** 2022-04-22

**Authors:** Linda Forst, Tessa Bonney

**Affiliations:** Environmental and Occupational Health Sciences, School of Public Health, University of Illinois Chicago, 1603 W. Taylor Street, #1045, Chicago, IL 60612, USA; tbonne5@uic.edu

**Keywords:** temporary staffing, temporary services sector, precarious work, occupational health surveillance, workers’ compensation, occupational injury, worker justice, occupational health equity, worker well-being

## Abstract

Temporary staffing has an increasing role in world economies, contracting workers and dispatching them to work for leasing employers within countries and across borders. Using Illinois as a case study, co-authors have undertaken investigations to understand the occupational health, safety, and well-being challenges for workers hired through temporary staffing companies; to determine knowledge and attitudes of temp workers and temp staffing employers; and to assess temporary staffing at a community level. Temporary staffing workers in Illinois tend to be people of color who are employed in the most hazardous sectors of the economy. They have a higher rate of injury, are compensated less, and often lose their jobs when injured. Laws allow for ambiguity of responsibility for training, reporting, and compensation between the staffing agency and host employers. Our findings illustrate the ways in which principles of fairness and equity are violated in temporary staffing. Shared responsibility for reporting injuries, providing workers’ compensation insurance, and training workers should be mandated in law and required in contractual language between temporary staffing and host/contracting employers. Monitoring, enforcement, and adjustment of the law based on experience are required to “promote inclusive and sustainable economic growth, employment and decent work for all.

## 1. Introduction

Since the end of World War II, temporary staffing has had an increasing and institutionalized role globally, employing workers and dispatching them to work in businesses that outsource jobs [1]. In this employment arrangement, the temporary staffing company is the employer of record—the temp company issues short-term labor contracts that cover wages, hours, and limited social benefits and lists details of the job [2]. “Host” employers “lease” workers from temporary staffing companies, assigning and directing job tasks at the site where the work is done, i.e., the host’s enterprise [3]. The temporary staffing work arrangement occurs both within countries and across borders [4].

In the U.S., temp workers are mainly supplied to the warehousing, manufacturing, and service sectors of the economy; the service sector includes, mainly, secretarial support, healthcare, and janitorial services [5]. Temp and host employers are legally required to follow labor regulations with respect to wages, hours, and insurance; they are also bound by occupational safety and health (OSH) regulations [6].

In the U.S., outside of healthcare, temp workers often earn significantly lower wages than directly hired workers doing the same jobs [7,8,9]. In contradistinction to other workers, temp workers are not provided with health insurance or paid sick leave and they have no job security or voice in the settings where they work [10]. Despite the legal mandate for joint responsibility of the temp and host companies in protecting temp workers, loopholes and violation of these laws are common and easy to obscure [1]. Supplementary laws have been put into place to assure temp workers the legal protections that apply to all U.S. workers and to prevent wage theft [7,8,9]. During the last decade, there have been reports of egregious health and safety violations leading to severe injury and death among temp workers [10,11]. The Occupational Safety and Health Administration, the U.S.’s enforcement agency, established the Temporary Workers Initiative to address this problem [12].

Illinois has the third largest temporary services sector in the U.S. With an overall population of 12.9 million and an employed population of 6.1 million, there are around 200,000 temp workers in Illinois, covering over 800,000 temp gigs, annually [13,14]. Worker advocacy groups have evolved since the early 2000s to support these workers [15].

Why is this issue important? As precarious work and its impact on the health, safety, and well-being of workers grows, studying temporary staffing can be instructive for occupational health advocates. Temp workers stand at the intersection of the most marginalized populations (immigrants, people of color, citizens returning from incarceration, recovering substance users, unskilled and low educated populations, and the urban and rural poor), dangerous jobs in the most hazardous sectors (laborers in manufacturing and warehousing), and the most precarious employment conditions (low wages, no social benefits, discharge from employment with no notice). The regulations and norms that protect most workers are not afforded temp workers. Consideration of the issues facing one of the most disadvantaged worker populations can guide investigation of their counterparts in other regions and in other precarious workforces. Moreover, the clear violation of standard occupational health and safety protections in temp work clarifies the pathway to justice and equity for all workers.

The co-authors of this report have undertaken a series of investigations to: (1) understand the occupational health, safety, and well-being challenges for workers hired through temporary staffing companies; (2) determine knowledge and attitudes of temp workers and temp staffing employers; and (3) assess temporary staffing at a community level. We present findings from our research on the temporary staffing sector and suggest actions to meet UN Sustainable Development Goal 8: Promote inclusive and sustainable economic growth, employment and decent work for all [16]. Approaches and findings in Illinois illustrate the ways in which the principles of fairness and equity are violated in temporary staffing; successful interventions in Illinois can be adapted to the conditions of temp staffing in other places.

## 2. Who Are Temp Workers? Where Do They Work and How Much Do They Earn?

There are over 250 registered day and temporary labor agencies with almost 730 branch offices in Illinois [17]. The number of individuals hired through temporary staffing is difficult to determine because it is counted differently by the various oversight parties. According to the American Staffing Association, there is an average of 245,700 temp workers each week in Illinois, with an annual employment of 1,265,500 [14]. In 2020, the U.S. Bureau of Labor Statistics (BLS) reported 181,330 temp workers in a quarterly census, comprising 3.2% of the total Illinois workforce (worker census was 5,627,660 in 2020 [18] USBLS, QCEW. https://www.bls.gov/cew/, accessed on 21 April 2022). The BLS reported 2,610,890 temp workers across the U.S. in 2020 (NAICS code 561320) [18].

As described in the introduction, temp workers in Illinois work, mainly, in the warehousing, manufacturing and service sectors. Table 1 depicts worker demographics in the occupations (job titles) and in the sectors that most commonly employ temporary workers. African American and Latinx workers are over-represented in most of these occupations, and women are overrepresented in office/administrative support and food preparation sectors [18].

Table 2 shows wage comparisons for the occupations most populated by temp workers in Illinois [7,18]. Median and hourly wages for temp workers, overall, ranged from 72–80% of the median, mean, hourly, and annual wages of workers who were hired directly in jobs in transportation and material moving and in production (manufacturing). In “cleaning” and “grounds maintenance” and business operations, temp workers made around 90% of the wages of directly hired workers. In food preparation and serving, temp workers made 106% of those of non-temp workers, though the wages in those occupations are extremely low. (For the US in 2020, the poverty level was $26,500 for a family of four) [19].

## 3. Temporary Staffing Employment at the Intersection of Race, Ethnicity, Immigration Status and Economic Hardship

Indigent persons in the U.S. are directed toward government employment programs with the goal of transitioning them out of poverty. Low skilled, low educated individuals, citizens returning from incarceration, and others who require government assistance are often placed into jobs through temporary staffing companies [20]. As shown above, individuals who have difficulty obtaining employment due to race, ethnicity, and immigration status are disproportionately employed in temp staffing. In the short term, placement in temp jobs has been shown to improve their odds of leaving “welfare” programs (i.e., becoming self-supporting); however, follow up data demonstrate a lack of long-term benefits and find that earnings of those placed in temp jobs may, in fact, diminish over time [21].

The co-authors collaborated on a series of studies that elucidate the connection of temporary staffing with poverty in high-economic-hardship areas of Chicago. The Greater Lawndale Healthy Work project consists of community-based participatory research in two contiguous urban neighborhoods that are mainly African American and Latinx [22]. North Lawndale, with a population of 34,794, is comprised mainly of African Americans (85%); it has a median household income of $28,327 with over 10,000 people living below the poverty line and 46.1% of households earning less than $25,000 [23]. South Lawndale, also known as Little Village, has a population of 71,399 and is comprised of 83.0% Hispanic/Latinx, 37.6% immigrants from Mexico and countries south of the US border; 12.3% of Little Village is Black, Non-Hispanic. The median income is $34,705 and 6210 households (34.4%) earn less than $25,000 [23]. Over 40% of Greater Lawndale residents have not graduated from high school and 39% do not speak English at home [23]. Combined, Greater Lawndale had an unemployment rate of 11.0% (prior to the COVID-19 pandemic) [23].

**Figure 1:**
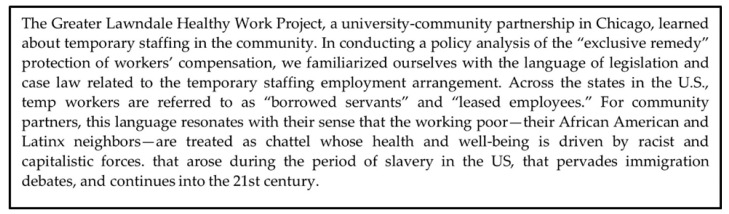
Community concerns that temporary staffing is rooted in racism.

Community researchers, trained by academic co-investigators, conducted a cross-sectional survey of 479 community residents from all parts of the neighborhood who self-identified as being precariously employed [24]. Of the 464 individuals who responded to the question about employment through temporary staffing companies in the prior 12 months, 190 (40.9%) reported that at least some of their paid hours came from temp agencies; 139 (30%) worked either most or all of their paid hours through temp staffing. Three-hundred and thirty-three reported having contacted an employment agency to find work in the prior 12 months. A landscape assessment of the area found five branch offices of temporary staffing companies located in Greater Lawndale [25]. Fourteen more branch offices are located in adjacent neighborhoods [17]. These are, logically, located where there are workers to hire. Figure 1 describes insights from community researchers who participated in the Greater Lawndale studies.

In collaboration with the Chicago Workers’ Collaborative, a worker center that advocates for temp staffing workers, Partners for Dignity and Rights carried out a matched pair testing study to measure and document patterns of discrimination against temp job applicants [26]. They identified a representative sample of 100 temporary staffing agencies in Greater Chicago—26 within the city limits, 43 immediately outside Chicago (non-Chicago Cook County), and 25 in adjacent counties. They paired six black and five Spanish-speaking testers to apply for jobs in these temp staffing companies. The testers applied for work using standardized procedures whereby a Latinx tester walked in first and applied, immediately followed by a Black tester, or vice versa. Table 3 is extracted from their report and demonstrates differences in timing of offer, type of work offered, adjusted wage rates, proximity of the worksite to the temp staffing office, the population demographics in the agency’s zip code, and the structure of the staffing audience. For every parameter, Latinx workers were offered more favorable outcomes than black workers.

## 4. Occupational Safety and Health in Temporary Staffing

### 4.1. Work-Related Injuries among Temp Workers

There are several studies demonstrating an association between status as a temp worker and increased risk of hazardous work: paced work, repetitive work, awkward postures, intensive use of vibrating tools and machinery, and lack of autonomy in applying skills at work [27,28,29,30]. Temp workers have lower self-rated health, report musculoskeletal symptoms of the upper extremity, and suffer from mental health conditions, such as depression [31]. Temp workers have a demonstrated increase in the rate of occupational injuries [7,8,9,27,32,33,34,35,36] and are less likely to return to work following an injury [37]. Temp workers in manufacturing had injury rates that were two to three times higher than their directly hired peers [36]. In a study we conducted of amputation injuries in Illinois workplaces from 2000–2007, we found 3984 work-related amputations; among the top ten employers, ranked in descending order by the number of amputations, half (five companies) were temporary staffing companies. One staffing company had six amputations, four of the whole arm or whole hand and two of legs [11]. Precarious workers in the Greater Lawndale study, described in the section above, reported a high prevalence of exposure to chemical, physical, ergonomic, and traumatic injuries, though identifying those who were currently employed through temp staffing was impossible to distinguish [24].

Occupational illness and injury surveillance in the US is problematic in the temporary staffing scenario. When a temp worker gets injured, it is the host company (where the worker is working) that reports any injury requiring more than first aid. By law, these injuries are listed on the OSHA log to be forwarded to OSHA at the end of the year [38]. Companies are sampled annually by the Bureau of Labor Statistics Survey of Occupational Illnesses and Injuries, the US’s national occupational health surveillance system (ref). There is no way to designate an injured worker as a “temp” employee; rather, temp workers count on the host’s roster. This makes it impossible to accurately determine injury numbers, rates, and trends for temp workers in the U.S. [39].

### 4.2. Workers’ Compensation

#### 4.2.1. How Workers’ Compensation Works in the Temp Staffing Scenario

Workers’ compensation is allocated via the insurance system and pays medical fees, lost wages, death benefits, and rehabilitation for people with work-related injuries. In the U.S., workers’ compensation laws and benefits vary greatly between the 50 states and three territories. In general, individual companies are assessed insurance rates based on the risk of injury for their employees. Logically, more hazardous economic sectors pay higher rates. Again, when a worker is injured on the job and requires more than first aid, the injury is reported on an OSHA injury log. In Illinois, if the worker loses more than 3 days of work, the employer is required to file a First Report of Injury in the workers’ compensation system. (Other states have different thresholds for filing a First Report of Injury). In the temporary staffing scenario, the temp staffing company is the employer of record and pays the workers’ compensation fees. However, work-related injuries occur at the host employer where the work is being done. As described above, the host is obligated to enter the injury on the OSHA log, but the temp staffing employer must report injuries to the workers’ compensation system; the temp staffing employer is responsible for medical coverage, lost work time fees, and the other work comp obligations. For a high number of injuries, the insurance companies (all private, in Illinois), increase insurance premiums based on the injury experience (“experience rating”) of a company.

Another important incentive for protecting workers is lost in the temp staffing arrangement. Since workers’ compensation is the exclusive remedy for a work-related injury, a worker may not sue an employer when a work-related injury occurs; rather, the worker applies for workers’ compensation to cover medical, indemnity (lost work time) and disability costs. Since the temp staffing company covers the workers’ compensation insurance, the worker gets compensation through the temp employer’s insurance policy, not through the host employer; yet the host employer is explicitly shielded from lawsuit by the exclusive remedy protection of workers’ compensation.

#### 4.2.2. Claims among Temp Workers

A higher incidence and severity of injuries was reported in several studies of temporary workers who receive pay per item produced (“piece work”) as opposed to pay per hour [33]. Moreover, temp workers had higher workers’ compensation claim rates [33]. In a study of Illinois workers’ compensation claims between 2007 and 2012, there were around 1500 claims per year filed by temp workers, most of them entailing lost work time [7]. This resulted in a litigated claim rate at around 1 claim per 100 workers [7].

The demographic characteristics of temp workers filing workers’ compensation claims from 2007–2012 is show in Table 4. Temp workers filing for workers’ compensation benefits are more likely to be male, single, have more dependents, be injured at a younger age, and earn about half the wages as compared to workers who are directly hired. Published data on workers’ compensation claims among temp workers in Illinois is not available beyond 2012. [Note that the number of employees is taken from the BLS quarterly census, which provides a snapshot in time, rather than the number of workers employed over a whole year. For most employment arrangements, the two are synonymous. With temp workers coming and going, the snapshot does not represent the total number of temp workers over a year. A better indicator would be temp worker X no. of jobs per year, but that would break uniformity and would not apply to the vast majority of workers].

#### 4.2.3. Consequences of the Disjointed/Irregular Workers’ Compensation Arrangement

First, as described above, work-related injury impacts workers who are hired directly by companies less often compared to those who are hired through temporary staffing companies. Injury rates are higher and affect single and younger individuals in temp work; temp workers earn lower wages and are therefore compensated by a lower amount in a workers’ compensation settlement. Second, in the immediate term, temp workers who lose time due to an injury generally end up losing their employment, since the host company is not obligated to maintain employment and the temp staffing company only employs workers on temporary contracts. Work-related injury or illness compounds the precariousness of workers hired through temporary staffing companies. Third, the host employer does not feel the impact of a work-related injury; there is no rise in insurance premiums, the host is protected from a lawsuit, and there is an ability to hide the temp status of injured workers. These conditions circumvent major incentives for providing a safe workplace for all workers. 

## 5. Health and Safety Perceptions

### 5.1. Temp Workers’ Perceptions of Health and Safety

In partnership with two Chicago-area workers’ centers, a team of graduate students and worker center members (known as Occupational Health Promoters) surveyed 98 temporary workers in 2015 to assess workers’ knowledge and perceptions of occupational safety and health (OSH) and to identify hazards encountered by temp workers on the job [40]. Temp workers surveyed in this study were largely employed in the warehousing and manufacturing sectors (51% and 43%, respectively) and were majority male (60%) and African American (97%). Study participants described a wide range of hazards encountered at host employer sites, particularly machinery and fall hazards; notably these hazards were the foci of OSHA Special Emphasis Programs at the time of survey deployment. Participants highlighted their lack of training around OSH issues and job task procedures prior to initiating work at a host employer’s site, and many indicated that the training they did receive was not applicable to the tasks that they were required to perform on the job. Several respondents explicitly noted that they were unsure as to whom they should report on-the-job safety and health concerns, highlighting the complexities of their employment situations and perceptions of employer responsibility for OSH.

### 5.2. Temp Employers’ Perceptions of Worker Health and Safety: Barriers to Meeting Legal Mandates

To gain input from employers, we conducted a “brainstorm” with 80 personnel from temp staffing companies in Illinois to determine their perceptions of the difficulty of protecting their employees in the temp staffing employment arrangement (unpublished). During a health and safety seminar conducted by OSHA in March 2020, attendees were invited to respond to the following prompt: “one of the ways addressing health and safety is challenging for temporary staffing companies is…” In the responses, they noted the following problems:

- Lack of control of the worksite by the temp staffing company
- Lack of knowledge of health and safety on the part of the host, the temp employer, and the worker
  o Workplaces have markedly different hazards from one another
  o There is a lack of expertise in delivering effective training
- Not following the rules related to training, reporting, and maintaining a safe workplace
  o The Training requirement is not followed because of cost of worker time for training (aggravated by high turnover, shift work, paying for time in training)
  o Injuries are not always reported
- Strained relationship between host and temp company
  o Temp workers are not treated the same as workers hired directly by the host
  o Host shifts blame to the temp staffing company
  o Host unwillingness to remediate a hazard even after an injury or a report from the temp company

Missing from this type of assessment is the perception of host companies in the temporary staffing arrangement. More widely investigating the perceptions of temp staffing employers and adding a survey of host companies’ perceptions could clarify their perceptions of the problems and potential solutions for protecting temp workers.

## 6. Limitations

The body of research presented in this manuscript was conducted largely by the co-authors and various research partners over time. Because of the laws pertaining to temp workers and their status (allowable contracts that do not clearly lay out shared responsibility of temp and host companies, deficient injury reporting, lopsided workers’ compensation responsibility), it is impossible to obtain accurate and comprehensive injury data on this workforce. Moreover, the fragmentation of labor contracts with poor oversight makes research investigations difficult to execute. Since these workers are employed in the most hazardous industries, we would expect them to have at least similar types and numbers of injuries as “permanent” workers. We have provided some evidence of higher and more severe injuries. Given the challenges of studying this fragmented workforce, we have tried to present our own, and others’ research to provide a 360-degree view of the challenges as regards health, safety, well-being, and equitable treatment of this workforce. Notably, the inability to identify injuries among temp workers makes it difficult to target interventions that would be preventive.

Safety climate—workers’ perceptions of their employers’ prioritization of safety—has been shown to be a leading indicator of injury. Safety climate is likely to be different between temp workers and directly hired workers in the same companies [41,42]. This phenomenon and its role in temp worker injuries requires further exploration.

Finally, this work comes from the United States, and mainly from one state (Illinois). Our review of scientific literature in other countries reflects similar issues [4,28,29,30,31,32,33,34,41]. Since policies are legislated locally, these findings are most likely to drive policy in Illinois and the US. However, other countries experience similar issues, and the approaches suggested here could guide research and interventions elsewhere.

## 7. Conclusions

There are several approaches that could be taken to address the disconnect between host and temporary staffing companies in appropriately protecting and compensating temp workers.

First, at a local (state) level, legislation should be enacted that lays out the issues that must be addressed in contractual language between the temp and host companies: (1) who is responsible for health and safety; (2) the conduct of safety audits of temp jobs; (3) training requirements for temp workers; (4) how the temp and host companies communicate about work-related illnesses and injuries as they occur. Language could be made available or mandated for inclusion in these contractual agreements.

Second, workers’ compensation legislation should be amended: (1) to reconsider how workers’ compensation insurance obligations are appropriated, such that responsibility is shared between the host and temp companies; (2) to create compensation equity between direct hires and temp workers by equalizing their indemnity (lost work time) compensation (i.e., raising temps’ rates to that of directly hired workers); (3) by removing workers’ compensation as the exclusive remedy for a work related injury in the case where a host company does not provide this insurance coverage (i.e., allowing a third party lawsuit).

Third, surveillance of occupational illness and injury in the temporary staffing arrangement needs to be adjusted. The OSHA illness and injury log should require reporting (i.e., add a data element) that differentiates temp vs directly hired employees. This would mark cases that could be counted in our national surveillance system (the BLS Survey of Occupational Injuries and Illnesses); it also would allow OSHA to better focus its health and safety consultation and enforcement activities.

Finally, we need a better understanding of the issues and needs of each of the players–first and foremost, workers, followed by temp staffing companies, host companies, and insurance companies. Their perceptions would help craft, implement and fine-tune interventions. Better policies, communication, and enforcement would ultimately lead to improved health, well-being, and equity of workers in the temporary staffing sector.

## Figures and Tables

**Table 1:** Proportion of women and minorities in commonly temp-staffed occupational categories in the US, 2020. From BLS Current Population Survey, 2020 Ref. [18].

Occupational Categories	Total Employed (in 1000 s)	Percent of Total Employed				
		Women	White	Black or African American	Asian	Hispanic or Latinx
Total employees ≥ 16 years	147,795	**46.8**	**78.0**	**12.1**	**6.4**	**17.6**
Transportation and material moving occupations	10,625	20.5	72.3	**19.4**	4.2	**23.9**
Production occupations	7590	28.3	77.8	**13.1**	5.6	**23.6**
Office and administrative support occupations	15,558	**72.7**	77.4	**14.3**	4.7	17.4
Building and grounds cleaning and maintenance occupations	5084	40.3	78.2	**14.2**	3.1	**37.9**
Food preparation and serving related occupations	6556	**54.4**	74.8	**13.9**	6.4	**27.3**

Note: temp worker employment is reported quarterly by the Bureau of Labor Statistics and exact numbers vary. Bolded values show over-representation of demographic groups in the particular economic sectors compared to the (top line) proportion of women, African Americans and Latinx individuals working in the US economy, overall.

**Table 2:** Comparison of wages of temp and non-temp workers in six occupations with highest number of temp workers, Illinois 2020 Refs. [7,18].

Occupation	# Temp Workers	# Non-Temp Workers	Temp/ Non-Temp	Temp Mean Hourly Wage	Non-Temp Mean Hourly Wage	Temp Non-Temp Mean Hourly Wage	Temp Mean Annual Wage	Non-Temp Mean Annual Wage	Temp Non-Temp Mean Annual Wage
Transportation/ Material Moving	83,500	581,380	14.4%	$14.40	$20.11	71.6%	$29,960	$41,830	71.6%
Production	29,230	405,280	7.2%	$15.53	$20.05	77.5%	$32,290	$41,710	77.4%
Office/Admin Support	17,410	760,560	2.3%	$18.30	$20.87	87.7%	$38,070	$43,400	87.7%
Business/Financial Ops	5840	345,970	1.7%	$35.94	$38.65	93.0%	$74,750	$80,390	93.0%
Food Preparation and Serving Related	1870	418,400	0.4%	$13.35	$12.63	105.7%	$27,770	$26,270	105.7%
Building/Grounds Cleaning/Maintenance	1530	161,000	1.0%	$14.45	$16.06	90.0%	$30,050	$33,410	89.9%

**Table 3:** Race-ethnic segregation of jobs offered to paired Black and Hispanic Testers. Ref. [26] Reprinted with permission from Brittany Scott at Partners for Dignity and Rights.

Jobs		Number of Jobs ^^				Segregated Jobs (Offered to Only One Tester)		Probability that Difference between % of Segregated Jobs and Zero Is Due to Chance
		All Jobs	Offered to Latinx Only	Offered to Black Only	Offered to Both	Jobs	% of Total Jobs	
**All Jobs**		**173**	**86**	**56**	**31**	**142**	**82.1%**	<0.00001 ***
**Timing of Offer**	At in-person application	123	53	41	29	94	76.4%	<0.00001 ***
	In follow-up messages	50	33	15	2	48	96.0%	<0.00001 ***
**Type of Work**	Factory 2nd or 3rd Shift	54	18	24	12	42	77.8%	<0.00001 ***
	Factory, Shift Unknown	17	11	5	1	16	94.1%	<0.00001 ***
	Factory 1st Shift	42	22	9	11	31	73.8%	<0.00001 ***
	Unknown	33	21	6	6	27	81.8%	<0.00001 ***
	Warehouse	23	12	9	2	21	91.3%	<0.00001 ***
	Other (Events, Cleaning, Food Service, Office)	21	13	8	0	21	100.0%	<0.00001 ***
**Adjusted ^** **Wage Rate** **($/Hour)**	$12.00–$15.00	63	32	18	13	50	79.4%	<0.00001 ***
	$11.00–$11.99 ^^^	46	24	14	8	38	82.6%	<0.00001 ***
	$8.00–$10.99	19	7	7	5	14	73.7%	<0.00001 ***
**Is Job Closer to One Tester?**	Latinx 3–21 Miles Closer than Black	67	23	13	31	36	53.7%	<0.00001 ***
	Distance is <3 Miles Different	44	19	16	9	35	79.5%	<0.00001 ***
	Black 3–16 Miles Closer than Latinx	19	9	8	2	17	89.5%	<0.00001 ***
**Population in Agency’s Zip Code**	>25% Black	20	6	9	5	15	75.0%	<0.00001 ***
	Neither (Non-Minority Neighborhood)	27	9	6	12	15	55.6%	<0.00001 ***
	>25% Latinx but not >25% Black	126	71	41	14	112	88.9%	<0.00001 ***
**Structure of Staffing Agency**	Single Office	26	17	6	3	23	88.5%	<0.00001 ***
	Multiple Offices in Illinois	69	30	28	11	58	84.1%	<0.00001 ***
	Offices in Multiple States	78	39	22	17	61	78.2%	<0.00001 ***

Based on 204 offers to testers during 65 applications by Black workers and 65 applications by Latinx workers or followup calls after those applications. ^ Adjusted by adding $0.25 per hour if offer mentions possible bonuses, raises, or earning from transporting other workers. ^^ Jobs offered to both are counted as one job. ^^^ Mean of all hourly wage rates offered is $11.64, median is $11.75. *** Difference between the number of jobs offered to Black workers and the number of jobs offered to both is statistically significant at <0.001.

**Table 4:** Characteristics of workers filing workers’ compensation claims in Illinois, 2007–2012 (Madigan, 2016).

	Workers Hired through Temp Agencies (*n* = 8936)	Direct Hire Employees (*n* = 303,263)	*p*-Value
Gender			<0.01
Male	6139 (68.7%)	198,885 (64.6%)	
Marital status			
Single	5015 (56.1%)	130,868 (43.2%)	
Married	3827 (42.8%)	168,458 (55.5%)	<0.01
Widowed/divorced	1 (0.0%)	219 (0.1%)	
Unspecified	93 (1.0%)	3718 (1.2%)	
Mean no. of dependents (s.d.)	1.3 (1.5)	0.9 (1.3)	<0.01
0	3993 (44.7%)	175,501 (57.9%)	
1	1625 (18.2%)	50,090 (16.5%)	
2	1569 (17.6%)	43,377 (14.3%)	
3	997 (11.2%)	21,872 (7.2%)	
4	496 (5.6%)	8397 (2.8%)	
5 or more	256 (2.9%)	4026 (1.3%)	
Mean age at accident (s.d.)	38.0 (11.3)	44.2 (11.7)	<0.01
Under 18 years	17 (0.2%)	988 (0.3%)	
18–24 years	1230 (13.8%)	16,658 (5.5%)	
25–34 years	2559 (28.6%)	53,563 (17.7%)	
35–44 years	2494 (27.9%)	79,469 (26.2%)	
45–54 years	1911 (21.4%)	94,558 (31.2%)	
55–64 years	579 (6.5%)	49,354 (16.3%)	
65 years and older	78 (0.9%)	6989 (2.3%)	
Attorney representation used	7974 (89.2%)	244,886 (80.8%)	<0.01
Weekly wage			
Mean (s.d.)	$420.35 (206.88)	$825.68 (466.92)	<0.01
Median	$367.20	$727.60	
Days between			
Accident and filing, mean (s.d.)	139.4 (305.2)	284.9 (573.9)	<0.01
Accident and filing, median	54.0	153.0	
Accident and decision, mean (s.d.)	688.3 (499.1)	841.9 (709.1)	<0.01
Accident and decision, median	558.0	689.0	

## Data Availability

Data are downloadable as described in the References section of this article.
